# Can rapid negative exclusion of blood cultures by a molecular method, enzyme template generation and amplification technique (Cognitor^®^ Minus), aid antimicrobial stewardship?

**DOI:** 10.1111/ijpp.12393

**Published:** 2017-08-18

**Authors:** Matthew Dryden, Agnes Sitjar, Zoe Gunning, Sophie Lewis, Richard Healey, Praneeth Satchithananthan, Natalie Parker, Taryn Keyser, Kordo Saeed, Helen V. Bennett

**Affiliations:** ^1^ Hampshire Hospitals NHS Foundation Trust Winchester UK; ^2^ School of Medicine University of Southampton Southampton UK; ^3^ Momentum Bioscience Long Hanborough UK

**Keywords:** Antibiotic resistance, Antibiotic Stewardship, Rapid diagnostics

## Abstract

**Objectives:**

Antimicrobial review is an important part of antimicrobial stewardship. A novel enzyme template generation and amplification technique (ETGA), the Cognitor^®^ Minus (Momentum Bioscience, Long Hanborough, UK) test, has a 99.5% negative predictive value for bacteraemia and fungaemia. This observational study asked two questions: (1) Does a negative ETGA, indicating no bacteraemia or fungaemia, aid antimicrobial review within 48 h of admission; (2) In this real‐life clinical setting, does a negative ETGA mean no bacteraemia or fungaemia?

**Methods:**

Consecutive blood cultures in patients with clinical infection were tested by ETGA. Negative results indicating an absence of bacteraemia or fungaemia were reviewed by the clinical infection team. Antibiotics were reviewed in these patients, and the role of the ETGA result in antibiotic change was recorded. Patients were followed up for a week.

**Key findings:**

A total of 197 of 246 samples gave a negative result by ETGA. This led to a positive stewardship outcome (antimicrobials changed) in 145 (73.6%) and negative stewardship outcome (empirical antimicrobials continued) in 47 (23.9%). Of the positive stewardship outcomes, the ETGA result supported the decision not to start antimicrobials in 21 (10.7%) patients, to stop antimicrobials in 21 (10.7%), to switch from IV to oral antimicrobials in 103 (52.2%) or to discharge or leave the patient at home in 58 cases (29.4%).

**Conclusions:**

Enzyme template generation and amplification supports antimicrobial stewardship decisions and may have cost advantages in reducing unnecessary empirical antibiotics and antifungal agents and in discharging patients from hospital earlier. ETGA result was consistent with blood culture findings and gave an earlier negative result.

## Introduction

Antimicrobial stewardship is an essential part of clinical care to reduce selection pressure on bacteria and reduce antibiotic‐associated complications.^1^ As such, it has become an important quality standard in health care, particularly in the UK.^2^ Approximately 10% of blood culture samples submitted for microbiological testing are found to be positive. The remaining 90% of samples are generally reported as negative after 5 days of incubation,^3^ during which time clinicians may continue to prescribe broad‐spectrum antibiotics to the patient with suspected bacterial infection. In addition, many patients with possible infection, for example neonates following complex deliveries, oncology patients with fever, elderly patients with deterioration who are admitted to medical assessment units and deteriorating patients on intensive care units (ICU), are treated empirically with intravenous antimicrobials and often for prolonged periods. This overuse of antimicrobials leads to increased pharmacy and clinical costs, the promotion of antimicrobial resistance and an increased risk of antimicrobial‐associated disease.

Enzymatic template generation and amplification (ETGA) (Cognitor^®^ Minus; Momentum Bioscience, Long Hanborough, Oxfordshire, UK) is a novel technology for the universal and rapid phenotypic detection of viable micro‐organisms by detecting the presence of bacterial or fungal nucleic acid‐modifying enzymes such as DNA polymerase.^4^, ^5^ This technology has been applied to blood culture using the ETGA *in vitro* diagnostic test. The ETGA test has been validated and CE‐marked, and it has a very high negative predictive value (NPV) for bacteraemia.^4^, ^5^ This previous study demonstrated a 99.5% NPV on blood culture sets measured after >12‐h incubation in the bioMerieux BacT/ALERT^®^ blood culture system, when compared with the result after 5 days. Its use in a routine clinical microbiology laboratory can determine patients who are not bacteraemic at a very early stage. Early detection of the presence or absence of bacteria or fungi in blood may help earlier review of empirical antimicrobial use and support antimicrobial stewardship programmes.

The ETGA test detects living organisms by separating intact (viable) organisms from the specimen and neutralising background levels of enzyme activity. Following microbial lysis, DNA polymerase activity is then detected using a proprietary synthetic DNA substrate that can be modified by DNA polymerase. ETGA can be used to universally detect any micro‐organism because DNA polymerase activity is common to all living things. ETGA does not identify micro‐organisms and does not detect microbial or human DNA or RNA. The amount of modified ETGA substrate is indicative of microbial DNA polymerase activity and can be measured by quantitative/real‐time PCR (qPCR). The assay has been validated for clinical use on the Cepheid Smartcycler.

This study assessed the clinical application of a validated diagnostic test using ETGA technology for the rapid confirmation and exclusion of negative blood culture specimens. The aim was to see whether early determination of negative bacteraemia and fungaemia allowed earlier antimicrobial review.

## Methods

### Subjects and setting

The study was carried out at two main hospital sites (the Royal Hampshire County Hospital, Winchester and the North Hampshire Hospital, Basingstoke) within the Hampshire Hospitals NHS Foundation Trust between December 2015 and May 2016. Patients with clinical infection who had blood cultures collected were included. This was an observational study which asked two questions: (1) Does a negative ETGA, indicating no bacteraemia or fungaemia, aid antimicrobial review within 48 h of admission; (2) In this real‐life clinical setting, does a negative ETGA mean no bacteraemia or fungaemia?

### Laboratory procedures

Blood was cultured and tested by ETGA (Cognitor^®^ Minus; Momentum Bioscience) from both the aerobic and anaerobic adult blood culture bottle (0.5 ml from each bottle), or from a paediatric blood culture bottle (1 ml), sent routinely to the microbiology laboratory. Samples were taken from blood culture bottles incubated on a blood culture machine (BacT/ALERT^®^, BioMerieux, Basingstoke, UK) for at least 12 h and that were negative at the time of sample collection. Briefly, in a Class II biosafety cabinet, the relevant volume of blood was withdrawn from the bottle and immediately placed in the ETGA sample tube. The tube was labelled with a working number which was linked to the laboratory identification number on the study worksheet. All blood culture bottles were returned to the incubator. The sampling step took no longer than 10 min. The sample was then processed in accordance with the ETGA information for use.^6^

Blood cultures which had signalled positive, indicating bacteraemia or fungaemia, were excluded. Prior to the main evaluation starting, a study was carried out to determine any risk of contamination by the sampling manipulation. Seventy consecutive blood culture bottles had samples extracted. Terminal culture was performed. No contamination was detected.

In practical terms to follow the routine working pattern of a clinical laboratory, the evaluation required extracting the sample from the blood culture bottle the morning after the blood culture had been received in the laboratory. Sequential samples were chosen with no other selection procedure. Culture of the blood continued even though a sample was removed for ETGA testing.

Negative ETGA results were interpreted as negative for bacteraemia and fungaemia. If the ETGA test gave a Ct value of ≤43.5, this indicated that the blood culture was not negative and the result was reported as ‘Not Determined’. The interpretation of such results in blood cultures which had so far shown no growth signal could mean that the microbes were in a growing phase and would signal positive at a later time, that the blood culture contained a non‐culturable microbe or that it was a false positive.

### Antimicrobial stewardship methods

#### Intervention

Enzyme template generation and amplification results were communicated by the laboratory to the clinical microbiology infection teams delivering patient consults at the two hospitals. The clinical microbiology teams consist of specialists and trainees in microbiology/infectious disease and antimicrobial pharmacists. The patients with negative ETGA results were clinically reviewed, and a clinical assessment was made on the contribution of the negative bacteraemia result to antimicrobial review at 12–24 h after commencing antimicrobials.

#### Outcomes and outcome measures

The clinical review was of history, examination, assessment of signs of sepsis, focus of infection, with investigations. Using criteria in antimicrobial stewardship protocols for antimicrobial review,^1^, ^2^ along with the ETGA result, the empirical antimicrobials were reviewed and decisions regarding antimicrobial changes were made by the clinical infection team reviewing the patient. The contribution of the ETGA result to the antimicrobial review was classified as ‘positive’ (empirical antimicrobials were changed or not started) or ‘negative’ (empirical antimicrobials were continued), and, if positive, whether antimicrobials discontinued or de‐escalated.

Patients were followed up by ward visits or telephone for a minimum of 5 days following antimicrobial review to determine the outcome of the review. The purpose of the follow‐up was for clinical review of the episode of infection and to ensure that there was no relapse or deterioration of the presenting infection.

#### Data management and analysis

The clinical review data were recorded on a spreadsheet, and as this was an observational evaluation, the data were not subjected to detailed statistical analysis. The organisation's research and development department reviewed the protocol of the evaluation. Ethical approval was not required as the blood cultures were collected routinely. No additional samples were collected or tested. The blood culture was tested by two validated licensed techniques – culture on the automated BacT/ALERT^®^ system and Cognitor^®^ Minus. As the latter was a validated and CE‐marked diagnostic method, it was appropriate for the result of this test to contribute to clinical decisions in the same way that inflammatory markers or bacteriology results might be used to form an opinion of the most appropriate therapeutic choice.

## Results

A total of 246 blood culture samples were tested by ETGA. The positive and negative controls run on each batch, gave expected results. One hundred and ninety‐seven (80%) samples gave a negative ETGA result and interpreted as true‐negative blood cultures. These were the patients who were followed up to see whether the result influenced antimicrobial decisions.

Of the 49 ‘Not Determined’ results, seven samples grew various microbes on culture. These were clear true positives. Forty‐two were negative by blood culture. It was not the remit of this study to establish why the ETGA and blood culture results were discrepant, but as these samples could not be called true negatives, they were excluded from the antimicrobial analysis.

There were two samples that were ETGA negative but grew bacteria. One grew a coagulase‐negative staphylococcus at day 5, this was regarded as a contaminant which may not have been present in the blood culture when the sample was removed for ETGA testing. One grew an anaerobic coccus on day 4. This organism failed to grow on subculture and was not identified. The focus was thought to be an intra‐abdominal source in an elderly patient with diverticulitis. This demonstrated that a negative EDTA equated to negative bacteraemia and fungaemia with a NPV of 99.5%, consistent with validation studies.^4^, ^5^

The suspected clinical focus of infection for the 197 patients followed up with consults is given in Table 1. One hundred and seventy‐five (88.8%) patients were on antimicrobials at the 24‐h consult which meant that the remaining 22 patients had not been started on empirical antimicrobials even though blood cultures had been taken.

**Table 1:** 197 patients with negative ETGA tests the day after blood cultures were collected

Suspected clinical focus	No.	No. on antimicrobials at consult	Negative outcome of ETGA result i.e. antimicrobials continued	Positive outcome	Antimicrobials not started	Empirical antimicrobials stopped	IV oral switch	Patient discharged
Urine	63	61	6	57	2	6	49	26
Chest	61	55	12	49	6	8	35	18
Skin/soft tissue	26	25	11	15	1	2	12	9
Intra‐abdominal/biliary	21	17	12	9	4	0	5	4
Endocarditis	1	1	1	0	0	0	0	0
Central nervous system	6	2	0	6	4	2	0	0
Unknown	19	14	5	9	4	3	2	1
Total (%)	197 (100)	175 (88.8)	47 (23.9)	145 (73.6)	21 (10.7)	21 (10.7)	103 (52.2)	58 (29.4)

There were many reasons why patients had had blood cultures collected but had not been started on empirical antimicrobials and this varied by focus. The clinical diagnosis was often not clear, and antimicrobials were withheld because the patient was not septic, but blood cultures had been collected. For example, in the intra‐abdominal category, antimicrobials had not been commenced in patients with pancreatitis, abdominal pain possible appendicitis or diverticulitis; in the central nervous system category because the infection was likely to be viral; in the chest category because there was suspected aspiration pneumonitis, viral infection, pulmonary oedema or the patient was unresponsive but not septic; in the unknown category because the patient had myeloma, lymphoma, neutropaenia, EBV or long vascular catheter *in situ*. It was regarded as a positive stewardship outcome (Table 1) if the ETGA result supported a decision not to start antimicrobials in a patient who had not received empirical antimicrobials.

The ETGA result had a positive stewardship outcome in 145 of 197 (73.6%) and negative stewardship outcome in 47 (23.9%). In the latter case, this meant that the empirical intravenous antimicrobials were continued as prescribed, despite the patient not being bacteraemic, and this decision was made on clinical grounds. Of the positive stewardship outcomes, the ETGA result supported the decision not to start antimicrobials in 21 (10.7%) patients, to stop antimicrobials in 21 (10.7%), to switch from IV to oral antimicrobials in 103 (52.2%) or to discharge or leave the patient at home in 58 cases (29.4%).

### Example #1

Mrs JR, 78, admitted from home to medical emergency unit, confused, fall. Cough and offensive urine. Temperature 37.8. Bi‐basal crackles in chest examination. Blood pressure 170/85, Heart rate 88, WBC 9.8 × 10^6^/l, blood cultures taken on admission. Commenced on intravenous piperacillin/tazobactam to cover chest and urinary sepsis.

#### Following day

Normal temperature, other observations stable. ETGA confirmed no bacteraemia on day 1. On post‐take review ETGA result supported de‐escalation of antibiotics from IV piperacillin/tazobactam to oral cotrimoxazole for 3 days and discharge on day 3. End patient diagnosis was chest infection.

The benefits of ETGA in this case resulted in reduction in intravenous antimicrobials, staffing costs and earlier discharge, as well as the benefits of improved antimicrobial stewardship, lower antimicrobial selection pressure and improved patient management.

### Example #2

Mr MC, 58, Intensive care, postoperative patient following abdominal surgery for carcinoma of the colon. Unstable postoperatively with higher oxygen and ionotropic requirements, and some renal derangement. Concern that physiological derangement represented early sepsis. Prophylactic antibiotics of amoxicillin, gentamicin and metronidazole were continued. Blood cultures were collected.

#### Following day

Physiological derangement improving. Procalcitonin and white cell count were within normal limits. ETGA confirmed no bacteraemia on day 1. ETGA results with the clinical assessment and inflammatory markers supported stopping antibiotics.

Enzymatic template generation and amplification result supported a reduction in intravenous antibiotics, with the benefits of improved antimicrobial stewardship, lower antibiotic selection pressure and improved patient management.

In the follow‐up of patients, no patient required restarting or escalation of antimicrobials when a decision had been made not to start, to stop or to switch from IV to oral. None of the 58 patients discharged were readmitted in the follow‐up period.

## Discussion

This study demonstrates that the ETGA test can be used to complement other laboratory parameters, to aid clinical review of antimicrobials in patient in hospital. A negative ETGA test with its high NPV equates to mean negative bacteraemia or fungaemia, and this information delivered at an early stage is helpful in the management of anti‐infective therapy. The ETGA result supports early antimicrobial review and equated to a true‐negative bacteraemia and fungaemia.

The strength of this study is in having an additional laboratory test, based on microbiological criteria, to complement physiological measurements to support therapeutic decision making. This is the first time that this use of this novel diagnostic technology has been reported in a real‐life clinical environment. The limitations of this study are that the antimicrobial review decisions, although based on published principles of antimicrobial stewardship, are nevertheless subjective clinical decisions of the individual clinician reviewing the patient and this is not a randomised controlled trial. However, by including two sites and a number of clinicians in the decision process, variations in individual practice and risk assessment were levelled out. There was no control group in this study, but as antimicrobial review is a subjective process anyway, it would be difficult to construct a randomised controlled to measure the effect of a laboratory test on antibiotic decision making. The ETGA test result was used as an additional piece of objective information, to complement all the other objective test results to make what is a subjective decision. A number of patients had a negative ETGA test but had a focal infection such as soft tissue infection or respiratory infection. These patients needed to continue antimicrobials, but often did not need intravenous antimicrobials.

Blood cultures are generally collected because a patient is clinically septic, has a fever, has deteriorated or to exclude bacteraemia.^3^ Patients with sepsis or suspected infection are usually treated with antimicrobials empirically. Although most blood cultures signal positive in the first 24–48 h, they are not reported as negative until 5 days of incubation is completed. While early antibiotics save lives, empirical treatment results in much overuse of antimicrobials and, if bacteraemia can be excluded, early antimicrobials may be discontinued or de‐escalated early (see patient examples above). We previously reported the use of a biomarker procalcitonin (PCT) to aid the decision to start or withhold antimicrobials in patients with suspected infection in the medical assessment unit or ICU.^7^ With a careful clinical assessment, we found the PCT result to be helpful in this decision. Our aim in this study was to see whether an additional objective test would help with antimicrobial prescribing decisions. ETGA, with a high NPV, fulfilled the criteria for a test that could exclude bacteraemia, and therefore most serious acute infection, early.

Clinical decisions made by doctors are made by analysis of a range of data streams: history, clinical examination, vital signs, radiology, laboratory tests, but are of necessity often empirical and subjective. Early confirmation of exclusion of bacteraemia is very useful. Patients who are clearly septic on clinical grounds will be treated appropriately. Patients with clear focal infection will be treated appropriately according to local guidelines for that focus. However, there are many patients who do not fall into clear categories and many of these patients receive antimicrobials unnecessarily and for too long. Exclusion of bacteraemia/fungaemia in such patients, with supportive biomarkers can provide useful reassurance to stop or avoid starting empirical antimicrobials. Such patients include some neonates and other paediatric patients,^8^ oncology and haematology patients with fever, elderly patients with deterioration who are admitted to medical assessment units and deteriorating patients on critical care units. There is such emphasis now on improving the quality of antimicrobial prescribing throughout the world^1^, ^2^, ^9^ and indeed, in the United Kingdom, there are now targets in the National Health Service to achieve this.^10^

In this study, the blood cultures chosen for testing with ETGA were sequential and not preselected. As a routine diagnostic tool, it may be best to employ the ETGA selectively. All patients put on empirical antibiotics or antifungals as a precaution, for example neonates with complications, oncology patients with fever, haematology patients with fever, deteriorating critical care patients should have blood cultures tested with ETGA. If negative, bacteraemia and fungaemia are highly unlikely and antimicrobials can be reviewed, discontinued or de‐escalated. Other patients selected by clinical review during the routine ward rounds could also be added to the list of ETGA tests if bacteraemia or fungaemia was part of the differential diagnosis but that diagnosis was unclear.

Enzymatic template generation and amplification is an effective and objective tool to aid the early review of empirical antibiotics and antifungals. It can lead to earlier reporting of confirmed negative blood cultures and provide additional information for recommending cessation or modification of antimicrobial therapy in patients at unclear risk of infection. This supports antimicrobial stewardship by: allowing early review of empirical antimicrobials, allowing discontinuation, de‐escalation or IV to oral switch or oral antimicrobials, allow a step down in patient care or hospital discharge, reduce antibiotic‐associated disease or reduce overall healthcare costs by reducing antimicrobial prescribing. ETGA may have cost advantages in reducing unnecessary empirical antimicrobials and antifungal agents and in discharging patients from hospital earlier. This was not formally assessed in this study and requires a health economic analysis to establish cost advantages for the use of ETGA.

## Conclusions

This is the first report of ETGA, a novel diagnostic technique, being used in a routine clinical environment to demonstrate the absence of bacteraemia or fungaemia at an early stage in the patients’ clinical care, and using the result alongside other laboratory parameters to support a clinical review of antimicrobial use. This observational study concludes that ETGA is a useful tool in antimicrobial stewardship.

## Declarations

### Conflict of interest

The Author(s) declare(s) that they have no conflicts of interest to disclose.

### Funding

This research received no specific grant from any funding agency in the public, commercial or not‐for‐profit sectors.

### Authors’ contributions

M.D. devised the project and drafted the manuscript; M.D. and K.S. analysed the data; M.D., S.L., R.H., P.S., N.P., T.K. and K.S. collected clinical data on ward rounds; A.S., Z.G.,H.B. carried out laboratory analysis.
